# Microwave-Responsive Metal-Organic Frameworks (MOFs) for Enhanced In Vitro Controlled Release of Doxorubicin

**DOI:** 10.3390/nano14131081

**Published:** 2024-06-24

**Authors:** Syeda Fiza Fatima, Rana Sabouni, Ghaleb Husseini, Vinod Paul, Hassan Gomaa, Remya Radha

**Affiliations:** 1 Department of Chemical and Biological Engineering, American University of Sharjah, Sharjah P.O.Box 26666, United Arab Emirates; 2 Department of Chemical and Biochemical Engineering, Western University, London, ON TEB 459, Canada

**Keywords:** metal–organic frameworks, MOFs in drug delivery, MOF synthesis techniques, MOF characterization, drug delivery, MOFs, cancer, doxorubicin, microwave

## Abstract

Metal-organic frameworks (MOFs) are excellent candidates for a range of applications because of their numerous advantages, such as high surface area, porosity, and thermal and chemical stability. In this study, microwave (MW) irradiation is used as a novel stimulus in vitro controlled release of Doxorubicin (DOX) from two MOFs, namely Fe-BTC and MIL-53(Al), to enhance drug delivery in cancer therapy. DOX was encapsulated into Fe-BTC and MIL-53(Al) with drug-loading efficiencies of up to 67% for Fe-BTC and 40% for MIL-53(Al). Several characterization tests, including XRD, FTIR, TGA, BET, FE-SEM, and EDX, confirmed both MOF samples’ drug-loading and -release mechanisms. Fe-BTC exhibited a substantial improvement in drug-release efficiency (54%) when exposed to microwave irradiation at pH 7.4 for 50 min, whereas 11% was achieved without the external modality. A similar result was observed at pH 5.3; however, in both cases, the release efficiencies were substantially higher with microwave exposure (40%) than without (6%). In contrast, MIL-53(Al) exhibited greater sensitivity to pH, displaying a higher release rate (66%) after 38 min at pH 5.3 compared to 55% after 50 min at pH 7.4 when subjected to microwave irradiation. These results highlight the potential of both MOFs as highly heat-responsive to thermal stimuli. The results of the MTT assay demonstrated the cell viability across different concentrations of the MOFs after two days of incubation. This suggests that MOFs hold promise as potential candidates for tumor targeting. Additionally, the fact that the cells maintained their viability at different durations of microwave exposure confirms that the latter is a safe modality for triggering drug release from MOFs.

## 1. Introduction

Cancer is one of the leading causes of death worldwide, with over 10 million deaths recorded in 2020 [1]. It is caused by the abnormal and uncontrollable growth of cells in the body that ultimately form a tumor and may metastasize to other body parts. When these tumors spread within the body, they infiltrate nearby healthy tissues and give rise to new tumors, referred to as malignant ones. The tumor can be surgically removed in the initial stages if accessible; however, cancer is often diagnosed in the advanced stages. Surgery is mainly coupled with radiotherapy or chemotherapy, as it is impossible to achieve single-cell accuracy [2]. Chemotherapy is one of the treatments used to combat this disease. Anticancer drugs called anthracyclines derived from the streptomyces bacteria family are used in chemotherapy to attack the DNA of rapidly growing cancer cells and inhibit their proliferation. Anthracyclines like adriamycin, doxorubicin, and daunorubicin work by causing cellular damage through the production of free radicals or by binding to the G-C base pairs in DNA, altering the nucleic acid structure. This disruption halts cell replication and division, ultimately leading to cell death [3,4]. There has been a record improvement in cancer survival rates due to anthracycline use. The extensive use of anthracyclines has dramatically improved cancer survival statistics globally. Prior to the 1960s, children diagnosed with leukemia had a 5-year survival rate of 30%. However, with the widespread adoption of anthracycline-based chemotherapy, the 5-year survival rate for children increased to 80% by 2010 [5].

Anthracyclines can circulate throughout the body, reaching tumors, but they may also impact healthy cells, resulting in unwanted side effects. This challenges the scientific community to develop better ways to administer the drugs while minimizing the damage to the patient’s body [3]. A crucial aspect of cancer drug delivery involves achieving the highest drug concentration at the tumor site. This necessitates using a carrier that prevents leakage in healthy cells, enables drug release at the target site, and maintains the drug’s stability. Nanomaterials are highly sought as carriers for anthracyclines due to their stability, size, and encapsulation efficiency, enhancing drug delivery to the tumor site. Additionally, their size-based accumulation at the tumor site is facilitated by tumor tissue’s enhanced permeability and retention effect. Certain nanocapsules have been developed, such as liposomes, micelles, carbon nanoparticles, and hydrogels. However, these systems come with limitations. Organic nanocapsules offer high biocompatibility but often lack the ability to precisely control drug release. On the other hand, inorganic systems enable controlled drug release, but they may not be as biocompatible and typically have lower drug-loading capacity. To address these challenges, Metal–Organic Frameworks, or MOFs, have emerged as promising candidates for drug delivery due to their properties that can ensure precise and sustained drug delivery. The main aim of using MOFs for drug delivery is to design drug carriers that show little or no toxicity in the body. The MIL (Materials Institute Lavoisier or Matériaux de l‘Institut Lavoisier) family of MOFs was pioneeringly employed for drug-delivery purposes.

MOFs are porous crystals composed of robust metallic clusters that establish strong bonds with ligands. Weaker bonds with organic linkers allow framework tunability and contribute to biocompatibility [6]. To enhance drug-release precision, stimuli-responsive drug-delivery systems (DDS) exploit the tumor microenvironment, boosting intratumoral accumulation. Among various stimuli, such as ultrasound and light, thermal medicine effectively achieves the desired therapeutic outcomes. Microwaves, for instance, generate localized heat within the tumor, increasing metabolic activity, drug uptake, chemical sensitization, and permeability. Certain MOFs exhibit responsiveness to diverse stimuli, whether external (e.g., temperature, light, magnetic field) or internal (e.g., pH and redox reactions). MOFs can be tailored for specific drug-delivery mechanisms. For example, some MOFs may incorporate organic and inorganic components to render them pH-sensitive. Under acidic conditions, protonation disrupts the coordination bonds, releasing the encapsulated drug. This property is beneficial in anticancer drug delivery because the tumor microenvironment is relatively acidic. Normal cells typically have a nearly neutral pH, whereas tumor cells, endosomes, and lysosomes exhibit a lower pH, ranging from 4.5 to 5.5 [7,8]. Several MOF-based stimuli-responsive drug-delivery systems have been reported in recent years. Iron-based MOFs are mostly chosen for drug-delivery applications due to their non-toxicity and biocompatibility [9]. These MOFs can be modified by integrating biological molecules in their structures to assemble Bio-MOFs with enhanced biocompatibility and stability [10,11]. In a study by Wyzogrozka et al., Fe-MIL-101-NH_2_ MOFs encapsulated an antituberculosis drug, achieving 12% drug loading. Cytotoxicity tests confirmed the safety of the drug-delivery system for administering antimycobacterial drugs in the lungs, indicating its suitability for tuberculosis treatment. Moreover, it showed potential as a contrast agent for MRI, enabling monitoring of the release process inside the body [12]. In a study by Jayanbakht et al., Cu-MOFs loaded with ibuprofen and coated with pH-sensitive carboxymethylcellulose (CMC) demonstrated low toxicity and stability against stomach pH, a crucial characteristic for oral drug-delivery systems used in treating gastrointestinal ailments [13]. Microwaves have the ability to improve the efficiency and precision of diagnostic modalities [14]. Their non-ionizing properties make them less harmful than conventional electromagnetic waves. The heat produced by microwaves can increase the tumor temperature uniformly, resulting in ablation, a valuable technique in thermal cancer therapy [15]. In one study, Jin et al. used microwave irradiation to induce changes to the host-guest bonding in carrier liposomes. Co-encapsulating DOX with NaCl enhanced the drug’s heat absorption property, allowing 67.6% drug release in 2 min with a 73.4% tumor inhibition rate [16]. Similar to liposomes, MOFs exhibit the desired properties for triggered drug delivery. Fu et al. synthesized Mn-doped MOFs and used MW irradiation to trigger drug release. This resulted in string elastic collisions on ions in Mn-ZrMOF nanocarriers. The MOF complex demonstrated responsiveness to microwaves, achieving a thermal conversion efficiency of 28.7% [17]. In a separate study, Yumei Bu and colleagues developed Fe_3_O4@ZnAl_2_O4:Eu^3+^@mSiO_2_–APTES for drug-delivery applications, harnessing the thermal responsiveness of ZnAl_2_O_4_:Eu^3+^. The study reported that 78.2% of the total drug was released in a controlled manner using on/off cycles of MW irradiation [18]. In a study by Chen et al., MW-responsive nanoparticles of mesoporous cobalt ferrite (H-mCo_x_Fe_3−x_O_4_) were used to encapsulate a chemotherapy drug called VP16. The MW enhanced release from 15% to 64% after seven exposure cycles. This outcome indicates that elevated temperatures are optimal for disrupting interactive forces, releasing the therapeutic agent in the process [19]. Since cancerous cells have a low-pH and high-temperature microenvironment, many acid-sensitive MOF pH-responsive MOFs are designed to treat cancerous cells responsive to the acidic pH. The ZIF family of MOFs is known to be highly pH-responsive as a carrier. Zhang et al. prepared a co-delivery system based on ZIF-90 to deliver doxorubicin (DOX) and 5-fluorouracil (5-FU) and investigated its stability in different pH values. X-ray diffraction (XRD) findings revealed that reducing the pH renders ZIF-90 less stable. Drug release in acidic conditions reached nearly 95% within 16 h [20]. Zhang et al. developed DOX-loaded Gd-MOFs and coated them with a glucose layer. The characterization and test results showed that under acidic conditions, DOX@Gd-MOFs-Glu exhibited successful release of DOX, as the glucose layer disintegrated due to the pH change [21]. In another study, Nejadshafiee et al. synthesized a magnetic BioMOF hydrothermally, using Curcumin as an organic linker [22]. Fe_3_O_4_@MIL-100 was investigated as a delivery agent for the anticancer drug doxorubicin hydrochloride (DOX). The highest DOX-loading capacity (∼19 mass %) was observed for MIL-100 composites containing about 16 mass % of Fe_3_O_4_ particles. The release profiles of the DOX-loaded carriers dispersed in phosphate-buffered saline (PBS, pH 7.4, 37 °C) indicate that the kinetics slow down after incorporating the Fe_3_O_4_ nanoparticles. The composites of Fe_3_O_4_ magnetic nanoparticles with MIL-100 MOF exhibit better DOX-loading capacity and controlled release kinetics than bare MNPs [23]. For the controlled release of doxorubicin hydrochloride, a bio-metal–organic framework (Bio-MOF) coated with a monodispersed layer of chitosan (CS; CS/Bio-MOF) was synthesized and applied as a pH-responsive and target-selective system for delivery of doxorubicin (DOX) in the treatment of breast cancer. The efficiency of the nanocarrier in loading and releasing DOX was assessed at different pH levels. According to the observations, the nanocarrier presents a slow and continuous release profile as well as a noticeable drug-loading capacity. Furthermore, the carrier exhibits pH-responsive and target-selective behavior by releasing a significant amount of DOX at pH 6.8, which is indicative of tumor cells and inflamed tissues. At higher pH values, it releases a substantially lower amount of DOX. In addition, the results indicated that pH is effective on DOX uptake by CS/Bio-MOF [24].

PH-responsive MOFs are frequently used for stimuli-responsive drug release in cancer therapy due to the acidic nature of the tumor microenvironment. In this paper, we have designed a system using external stimuli-responsive MOFs for cancer therapy, bearing in mind the sensitivity of MOFs to pH and the acidity of the tumor microenvironment (TME).

Several studies in the past have investigated the application of metal-organic frameworks for drug delivery and anticancer therapy, including the examples stated above; however, to the best of the authors’ knowledge, there is a scarcity of studies exploring microwave irradiation as an external stimulus for releasing doxorubicin from MOFs. To achieve this objective, we studied DOX’s encapsulation efficiency and capacity by Fe-BTC and MIL-53(Al) MOFs. Furthermore, we investigated doxorubicin’s maximum and cumulative release from both MOFs under microwave irradiation at 2450 MHz. Also, we conducted several characterization tests, including pristine and encapsulated MOFs before and after microwave irradiation, to understand the loading and release mechanisms. This in-depth analysis provides unique insights into the loading/release mechanisms that have not been explored in detail, particularly for MOFs as a heat-responsive nanocarrier for MW irradiation, thus demonstrating the potential promise of MOFs in advancing drug delivery and therapeutic applications. Aluminum is an affordable and abundant metal, making it suitable for the mass production of MOFs for various applications. Aluminum-based MOFs are popular due to their exceptional stability, biocompatibility, and large porosity, making them ideal for a wide range of drug-delivery applications [25,26]. Similarly, iron-based MOFs are popular for biomedical applications due to their thermal and mechanical stability, tunability, large surface area, and small particle size [27]. Iron-based MOFs are favored for drug-delivery applications due to their biocompatibility. This research aims to explore MOFs, specifically Fe-BTC and MIL-53(Al), as potential nanocarriers for doxorubicin, employing precisely controlled release methods through microwave irradiation under different pH conditions (5.3 and 7.4).

## 2. Materials and Methods

### 2.1. Materials

All materials in this study were of analytical grade and were used without further modification or purification. Fe-BTC and MIL53 (Al) were purchased from Sigma-Aldrich (supplied through LABCO LLC. Dubai, United Arab Emirates) under the trademark “Basolite ^®^ F300” and “Basolite^®^ A100”. For pH adjustment, 1 M hydrochloric acid (HCl, 37%) and sodium hydroxide (NaOH, 98%) were used. Doxorubicin hydrochloride (DOX) was procured from Euroasia Trans Continental (Mumbai, India). Phosphate-buffered saline (PBS) tablets from Sigma-Aldrich were employed to prepare 0.1 M PBS solutions. For MOF regeneration, ethanol (99.8%, Sigma-Aldrich) was used as the solvent. The reactive oxygen species (ROS) experiments utilized 3,3′,5,5′-Tetramethylbenzidine (TMB) (≥98% TLC) and dimethyl sulfoxide (DMSO) (≥99.7%) as key reagents.

### 2.2. Methods

A comparative study of the release of DOX using Fe-BTC and MIL-53 MOF is reported with and without MW irradiation. For the release experiments, a UWave-2000 Multifunctional Microwave equipped with a 2450-MHz microwave generator was used to produce microwaves at 200 W. A MIKRO 220-Hettich centrifuge (HettichLab, Tuttlingen, Germany) was employed to separate the loaded MOF samples for further analysis. DOX concentrations were determined using a UV–Vis spectrophotometer instrument (Peak instrument, UV-9200/VIS-7220G, Houston, TX, USA) in the 200–600 nm wavelength range. A calibration curve was generated based on the absorbance of DOX at its characteristic peak of 480 nm. In case of requiring sample drying, a Venticell Oven (MMM Group, Munich, Germany) was used at a temperature of 80 °C. Dynamic light scattering was employed to measure the average particle size distribution using DynaPro NanoStar (Wyatt Technology, Santa Barbara, CA, USA).

### 2.3. Characterization Tests

The loading efficiency of the MOFs and the systemic release mechanism are investigated using various characterization techniques, including FTIR (Fourier Transform Infrared Spectroscopy), XRD (X-ray Diffraction), Field Emission Scanning Electron Microscopy (FE-SEM), and TGA (Thermogravimetric Analysis). Also, the effects of different stimuli, such as pH and microwave irradiation, on the release of DOX from MOF samples are investigated. The encapsulation and release efficiencies were measured using a UV-spectrophotometer. Before and after the encapsulation of DOX, the MOF samples were characterized using several techniques. First, the crystal structure was verified using X-ray diffraction (XRD) measurements. In this work, XRD patterns of the samples were collected on a Bruker D8 ADVANCE system (Bruker, Billerica, MA, USA) with a Cu tube and a linear detector (LYNXEYE XE, Stockholm, Sweden). The measurements were performed over a 2θ range of 5.0–60° with a step size of 0.03°. The shapes and crystallite morphologies were examined using Thermo Fisher Scientific Apreo C FE-SEM model, Brno, Czech Republic. FTIR spectra were obtained on an FTIR spectrophotometer (PerkinElmer, Waltham, MA, USA) using the transmission KBr pellet (pressed-disc) technique, operating in the range of 4000 to 450 cm^−1^, and 10 scans were signal-averaged with a resolution of 1.0 cm^−1^. Thermal gravimetric analysis (TGA) of the MOF samples was obtained using a Pyris 1 TGA instrument (PerkinElmer, Waltham, MA, USA) at a heating rate of 20 °C·min^−1^ from 25 °C to 800 °C.

### 2.4. Reactive Oxygen Species (ROS)

To detect ROS, a mixture containing 3000 µg of 3,3′,5,5′-Tetramethylbenzidine (TMB) in 3 mL DMSO along with 3000 µg of Fe-BTC or MIL-53(Al) and 60 mL hydrogen peroxide (H_2_O_2_) was used. These components were dissolved and then irradiated with a MW apparatus for 20 s with an outpower of 200 W and a frequency of 450 MHz. Afterward, the mixture was centrifuged at 6000 rpm for 10 min, and the supernatant was analyzed. UV–Vis absorption spectra were collected to quantify the ROS generated as a result of MW exposure.

### 2.5. DOX Encapsulation and In Vitro Release

**Figure 1:**
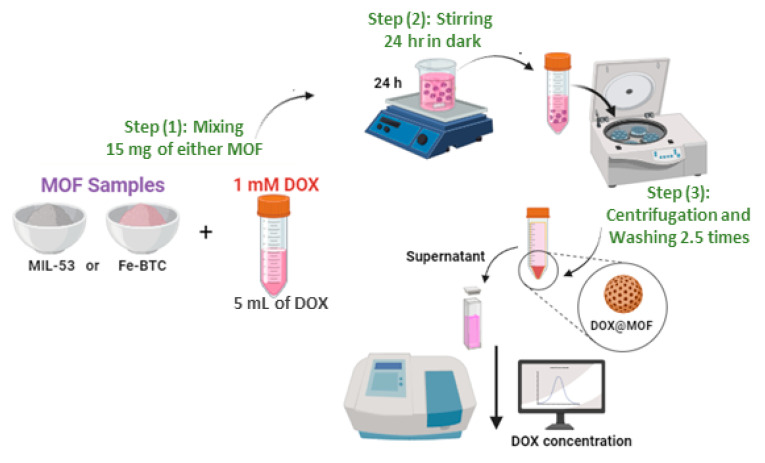
Doxorubicin encapsulation procedure.

DOX loading experiments were carried out in triplicates. In each run, 15 mg of the MOF sample (Fe-BTC and MIL-54 (Al)) was mixed with 5 mL of 1 mM DOX solution (with deionized water (DI) as the solvent). The mixture was allowed to stir overnight under dark conditions for 24 h. The resulting suspension was centrifuged for 15 min at 6000 rpm. The supernatant was removed, and the absorbance was measured in a UV–Vis spectrophotometer to determine the concentration of the DOX solution. The resultant material was washed twice with PBS to remove any unencapsulated DOX and then dried in an oven at 80 °C for 1 h (Figure 1).

To calculate the encapsulation efficiency and capacity of the MOF samples, Equations (1) and (2) were applied [24,28,29].

(1) $$\mathrm{Encapsulation}\mathrm{efficiency}\;(\%)=\left(\frac{C_{o}-C_{F}}{C_{i}}\right)$$

(2) $$\mathrm{Encapsulation}\;\mathrm{capacity}\;(\mathrm{wt}.\;\%)=\left(\frac{m_{loaded}}{m_{loaded}+m_{MOF}}\right)\times 100\;$$

where *m*_*loaded*_ is the mass of DOX (mg) and *m*_*MOF*_ is the mass of the MOF before loading (mg). *C*_o_ is the initial DOX concentration (mM) before loading and *C_F_* is the final DOX concentration (mM) after loading in the supernatant.

For the release experiments, 15 mg of the DOX-loaded DOX@Fe-BTC or DOX@MIL53 (Al) was introduced into 5 mL of 0.01M PBS at two pH levels of 7.4 and 5.3 and maintained at 37 °C. These experiments were conducted with and without exposure to MW using the UWave-2000 Multisector station. At specific time intervals of 2.5 min of MW exposure at a frequency of 2450 MHz and 200 W, the samples were centrifuged, and an aliquot of the supernatant was withdrawn for spectral analysis and replaced with an equal amount of fresh PBS. Following this, the release percentage was calculated using Equation (3) [30].

*Cumulative release efficiency* (%) = ∑(*C_i_*/*C_max_*) (3)

where *C_i_* represents the DOX aliquot concentration (mM) at each time point and *C_max_* is the DOX’s maximum release concentration (mM), as determined through spectroscopic analysis of the supernatant collected from the sample.

### 2.6. Microwave Treatment and Cytotoxicity Assays

For the in vitro cellular studies, approximately 20,000 cells in 6 mL media were placed in sterile 15 mL tubes and subjected to microwave treatment. The microwave oven was programmed to emit radiation such that the temperature of the sample did not exceed 42 °C. The sample temperature was raised in steps and a temperature sensor was placed inside the 15 mL tube to monitor the temperature of the sample. In the first step, the microwaves irradiated the sample to raise the temperature from room temperature to 29 °C in 3 min; then it was increased to 36 °C in another 3 min, and finally to 38 °C in 2 min after which it was held for 3 more minutes. Concurrently, control tubes (untreated cells and cells + MW) and DOX-loaded MIL-53(Al) were prepared at a 5 µg/mL concentration. Following the microwave treatment, approximately 1.5 mL of the treated cell culture solution was aseptically transferred into a 24-well cell culture plate in triplicates. After a 24 h incubation period, the MTT assay was conducted to assess cell viability and to evaluate the effects of the microwave treatment on the cells and its impact on DOX release from MIL-53(Al) concerning the viability of Jurkat cell lines.

For the cell viability assay, after the 24 h incubation, MTT (3-(4,5-dimethylthiazol-2-yl)-2,5-diphenyltetrazolium bromide) was added to each well, maintaining a concentration of 0.5 mg/mL, and the plate was further incubated at 37 °C for 4 h. After this incubation period, the culture solutions from each well were carefully collected into separate 2 mL Eppendorf tubes and subjected to centrifugation at 500× *g* for 4 min. The supernatant was delicately discarded, and approximately 500 µL of DMSO was added to each tube to dissolve the formazan crystals that had accumulated as pellets. These solutions were thoroughly mixed, placed in the dark for 10 min, and then transferred to 96-well plates. The optical density (OD) of each well was measured at 570 nm using a microplate reader.

To determine cell culture vitality, the ratio of the mean OD values of the treated cells to the mean OD of the control (untreated cells) was calculated and then multiplied by 100%. This calculation provided a quantitative assessment of cell viability and the impact of microwave-assisted treatment on the cells. Cell culture viability was determined using the following formula:

(4) $$\%\;Culture\;viability=\frac{mean\;absorbance\;of\;test\;wells}{mean\;absorbance\;of\;control}\times 100\%$$

#### Cytotoxicity Studies with Free MIL-53(Al) MOFs

A standard MTT plate assay was conducted to assess biosafety and to confirm the non-toxicity of using MOFs (MIL-53(Al)) in human cell lines. Approximately 10,000 Jurkat cells were seeded into the wells of a 96-well microplate. Various concentrations ranging from 0 to 625 µg/mL of free MIL-53(Al) MOFs were introduced into the wells, followed by a 48 h incubation in a cell culture incubator. Subsequently, an MTT assay was performed as per the procedure outlined in Section 2.4 and the viability of the cells under each condition was determined.

## 3. Results and Discussion

### 3.1. Characterization Tests

#### 3.1.1. Morphology

**Figure 2:**
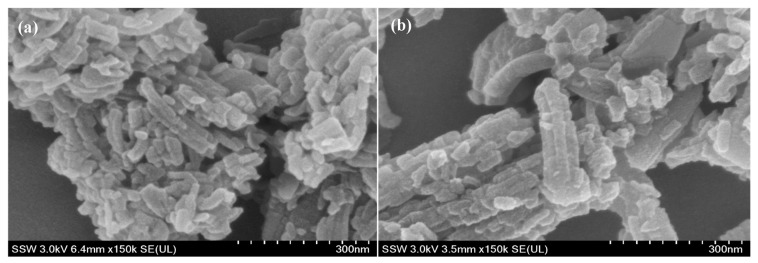

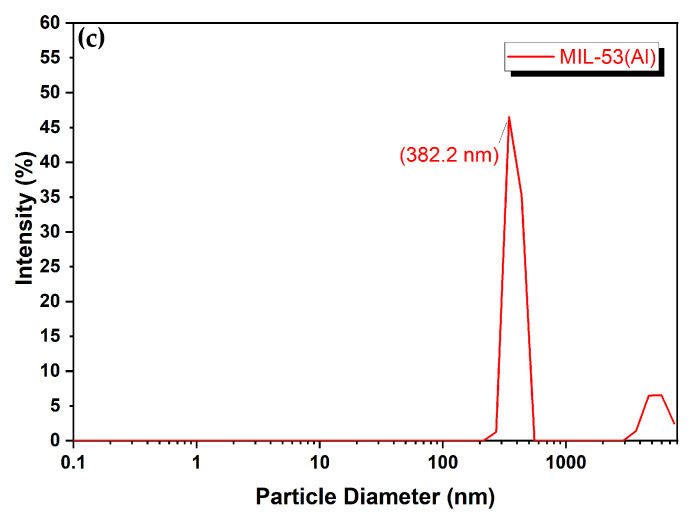
FE-SEM images of MIL-53: (**a**) MIL-53; (**b**) MIL-53 after loading with doxorubicin; (**c**) particle size distribution of Fe-BTC.

The crystal morphology and sizes of the MIL-53(Al) samples were observed by Field Emission Scanning Electron Microscopy (FE-SEM), as shown in Figure 2a,b. As shown by the images, MIL-53(Al) shows clustered rod-like crystals of 100–200 nm in size. Nanoparticles in the range of 100–400 nm in size can freely pass through large pores and passively target the tumor owing to the enhanced permeability and retention effect and because the maximum size of vascular pore cutoff size can reach up to µm sizes [31,32,33,34]. However, this criterion depends on several factors, such as the organ being targeted, the size of the fenestrations of the vasculature in that organ, the type and location of the tumor, and the method of nanocarrier administration (intravenous injection, tumor injection, oral, etc.) [35,36,37]. Furthermore, Figure 2b indicates the stability of MIL-53(Al) for drug delivery as it retains its rod-like structure post-DOX loading with no significant change to crystal morphology or size [38,39,40].

**Figure 3:**
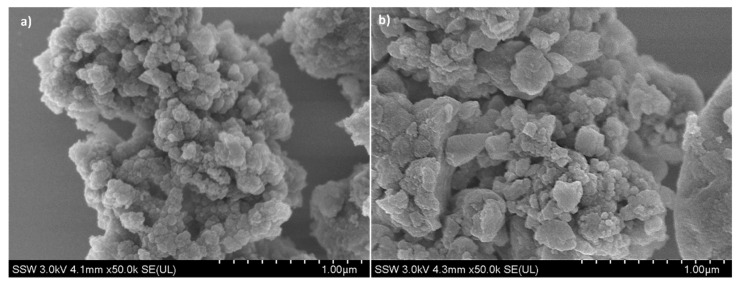

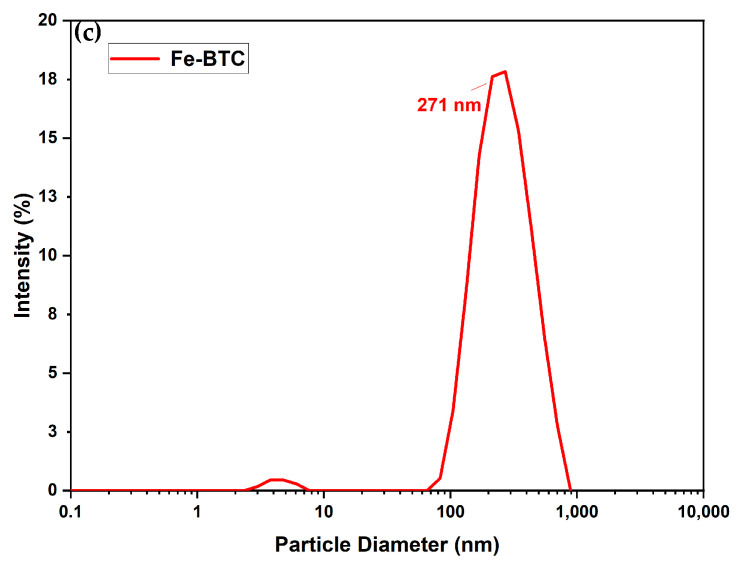
FE-SEM images of Fe-BTC: (**a**) Fe-BTC; (**b**) Fe-BTC after loading with doxorubicin; (**c**) particle size distribution of MIL-53.

Figure 3a,b represent the FE-SEM images of Fe-BTC samples before and after DOX loading, respectively. Irregular spherical agglomerates were observed, which is commonly attributed to the low degree of crystallinity of pure Fe-BTC [41]. The low crystallinity indicates disorder in the atomic arrangement, leading to non-uniform particle shapes. The morphology was retained post-loading without causing any significant alteration to the external crystal structure, indicating DOX was encapsulated inside the pores and not visible on the external surface [41,42,43,44,45]. Dynamic light scattering was performed to confirm particle size further, as shown in Figure 2c and Figure 3c. Also, narrow size distribution indicates good dispersity [25]. The results confirm that MIL-53 and Fe-BTC have particle sizes of ~477 nm (with around 83% of the particles having around 382.2 nm particle diameter) and 270 nm, respectively, which are desirable for our application [30,46].

#### 3.1.2. X-ray Diffraction (XRD)

**Figure 4:**
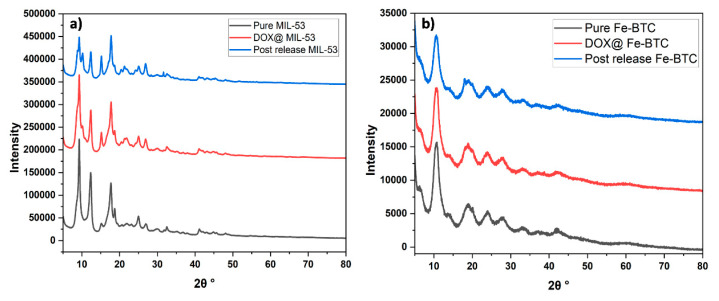
XRD patterns of (**a**) MIL-53, DOX@ MIL-53, and DOX@MIL-53 post MW release; (**b**) Fe-BTC, DOX@Fe-BTC, and DOX@Fe-BTC post MW release.

The XRD pattern of the pure MIL-53(Al) is presented in Figure 4a, which follows the distinctive characteristic peaks at 2θ values of 9.28°, 12.42°, 17.8°, 25.14°, 27.08°, as reported in the literature [47,48]. The sharp, well-defined peaks observed in the XRD pattern of MIL-53(Al) are indicative of its high crystallinity compared to the after-DOX-loading and post-release samples. Despite the minor changes in the intensity of the XRD pattern for the after-loading and post-release samples, MIL-53(Al) exhibits excellent structural stability. Further, the observed reduction and weakening in the XRD peak intensities in both the after-loading and post-release samples can be attributed to the successful encapsulation of DOX molecules within the pores of MIL-53(Al) [49].

In contrast, the Fe-BTC XRD pattern revealed low crystallinity characteristics, evident in the broader and less defined peak patterns (Figure 4b). The XRD pattern exhibits clear peaks at 2θ values of 10.72°, 18.9°, 24.12°, 28.14°, and 33.52°, consistent with the reported literature for Fe-BTC MOF [50,51,52]. The characteristic peaks were preserved after loading and post-release, proving the structure’s stability.

#### 3.1.3. Thermal Gravimetric Analysis (TGA)

**Figure 5:**
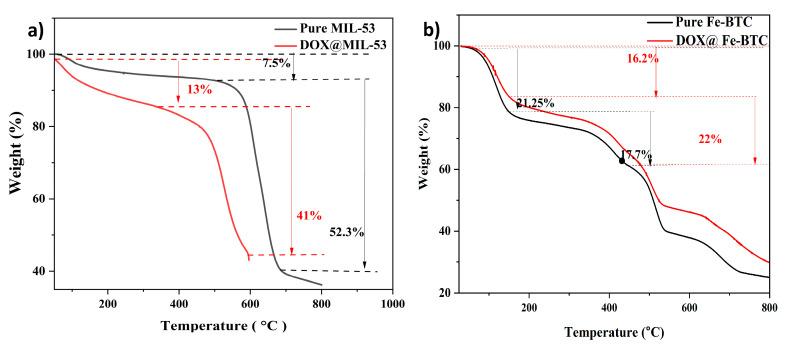
Thermal gravimetric analysis (TGA) curves of (**a**) MIL-53 and (**b**) Fe-BTC before and after loading with doxorubicin. Dashed and dotted lines represent the weight loss %.

The thermal stability of Fe-BTC and MIL-53 (Al) was investigated over the 30–800 °C temperature range by thermal gravimetric analysis (TGA). Figure 5a depicts the thermal stability of MIL-53 with two main weight-loss stages. The first weight loss of 7.5 wt.% from 50 to 450 °C corresponds to the desorption of solvents and free water molecules entrapped within the MOF pores. Beyond 450 °C, a substantial extra weight loss (~52 wt.%) was observed, mainly associated with the framework, particularly ligand decomposition [53,54]. The elevated decomposition temperature observed for MIL-53(Al) is consistent with the literature and is well-suited for drug-delivery applications [40]. A similar trend was observed for the DOX@MIL-53(Al) samples, proving that the MOF’s stability was maintained following drug loading.

The results depicted in Figure 5b illustrate the thermal stability behavior of Fe-BTC as indicated by its TGA. For pure Fe-BTC, around 21% of decomposition occurs between 70 and 150 °C. In the loaded MOF, this decomposition range slightly shifts to 60–150 °C. According to the literature, the weight loss observed between 50 and 100 °C for Fe-BTC is due to the evaporation of water molecules trapped in the MOF’s pores [55]. Additional weight loss is noted after 350 °C, attributed to the calcination of the MOF’s organic components [56].

#### 3.1.4. N_2_ Adsorption/Desorption Isotherms

**Figure 6:**
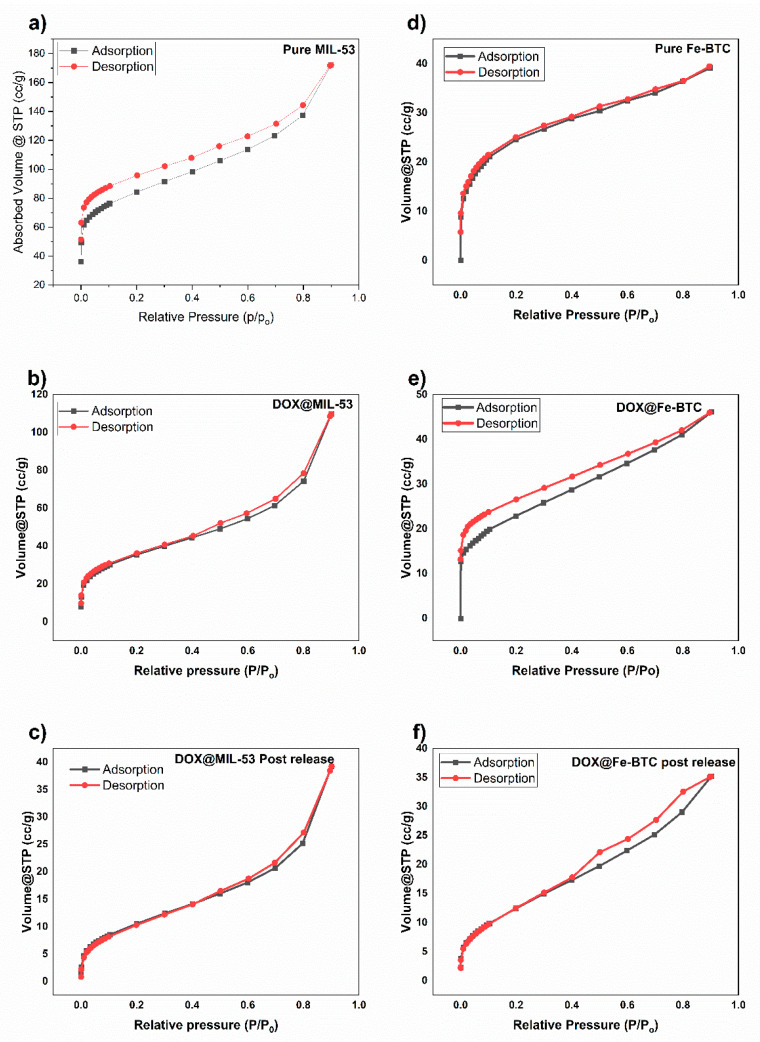
The N_2_ adsorption/desorption isotherms for (**a**) MIL-53, (**b**) DOX@MIL-53, (**c**) DOX@MIL-53 post release, (**d**) Fe-BTC, (**e**) DOX@Fe-BTC, and (**f**) DOX@Fe-BTC post release.

N_2_ adsorption–desorption experiments were used to characterize the surface area of MIL-53 and Fe-BTC, as shown in Figure 6a–f. The structural properties of MIL-53(Al) and Fe-BTC MOFs are summarized in Table 1.

The surface area and pore distributions were obtained using the Brunauer Emmett Teller (BET) method. The specific surface area of pristine MIL-53(Al) was 329.74 m^2^/g with a pore volume of 0.242 cm^3^/g. These values reflect the highly porous nature of MIL-53(Al). In the case of Fe-BTC, the determined surface area was 247.8 m^2^/g with a pore volume of 0.168 cm^3^/g. While slightly lower than those of MIL-53(Al), these values still indicate a substantial degree of porosity. An important finding is the observed decrease in surface area and pore volume for both MOFs following the encapsulation of DOX. This reduction can be attributed to the presence of entrapped DOX molecules within the MOF pores, which partially occupy the available pore space [28,30]. The isotherm shape observed was Type IV, indicating that adsorption on mesoporous solids occurs through multilayer adsorption due to capillary condensation [57]. A hysteresis loop was observed, which is associated with capillary condensation in mesopores.

#### 3.1.5. Fourier Transform Infrared (FTIR)

**Figure 7:**
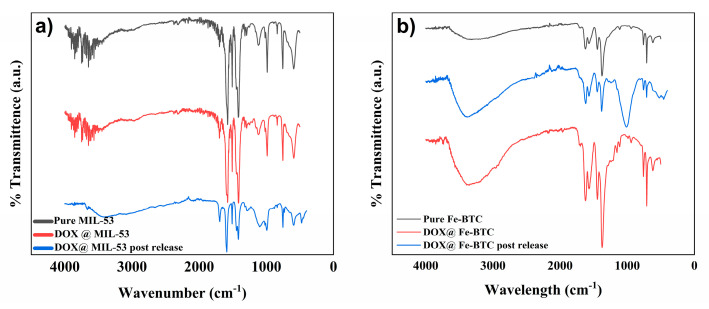
FTIR spectra of (**a**) MIL-53 before loading, after loading, and post-release; (**b**) Fe-BTC before loading, after loading, and post-release.

The FTIR analysis presented in Figure 7a shows the absorption peaks of MIL-53(Al) before and after DOX loading. The measured IR spectrum of MIL-53(Al) supports previously reported results in the literature [48,58]. The absorption peaks at 1417 cm^−1^ depict C-O-H stretching, whereas the peaks at 1697 and 1576 cm^−1^ reveal the presence of COO– asymmetric bond stretching vibration. The absorption peak at 3526 cm^−1^ relates to the ·OH stretching vibration [48]. The FTIR spectra of the DOX@ MIL-53(Al) showed peaks that were similar to the pristine MIL-53(Al) but were slightly higher and wider, indicating the encapsulation of the drug molecules in the MOF [30].

Figure 7b presents the FTIR spectra of Fe-BTC and DOX@Fe-BTC particles. The broad peak in the range 3000–3800 cm^−1^ is attributed to the presence of trapped water molecules in the Fe-BTC pores. Moreover, the peaks in the range 1000–3000 cm^−1^ identify the BTC organic linker. The peaks at 1445, 1567, and 1622 cm^−1^ signify the symmetric/asymmetric carboxylic stretching vibration [59]. Additionally, the peak at 1377 cm^−1^ suggests the C–O bond from the organic linker [45]. On the other hand, the peaks in the range 800–500 cm^−1^ are associated with the metal cluster in the MOF structure. The peaks at around 760 and 711 cm^−1^ are attributed to the Fe–O bond stretching. In Fe-BTC-DOX particles FTIR spectrum, the peaks at 1620 cm^−1^ reveal the carboxylic acid C=O bonds corresponding to the encapsulated DOX [60,61,62].

#### 3.1.6. Energy Dispersive X-ray Analysis (EDX)

**Figure 8:**
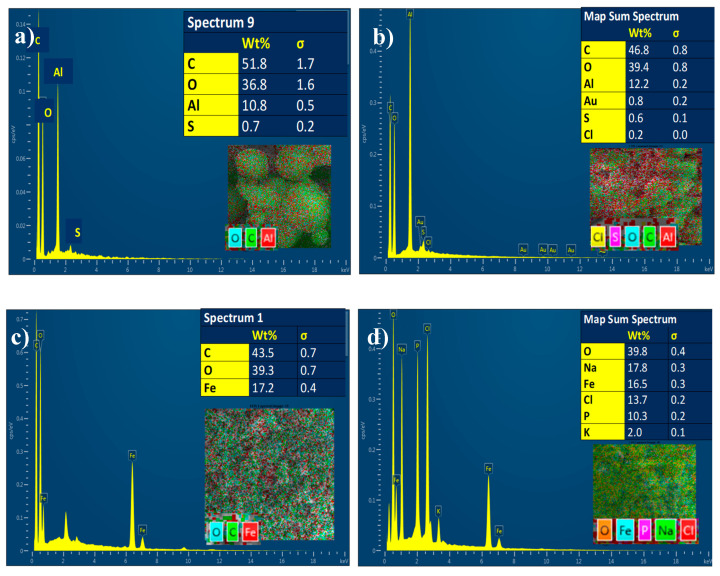
EDX of (**a**) MIL-53, (**b**) DOX@MIL-53, (**c**) Fe-BTC, and (**d**) DOX@Fe-BTC.

The chemical composition of MIL-53(Al) was determined using energy-dispersive X-ray analysis (EDX). The presence of C (51.8%), O (36.8%), and Al (10.8%) in the Al-MOF was confirmed, as demonstrated in Figure 8a. The presence of the Fe (17.2%) metal ions was detected for the Fe-BTC along with C (43.5%) and O (39.2%) samples (Figure 8c), and the DOX loading was confirmed by the detection of Cl element, which is attributed to DOX.HCl, as shown in Figure 8b,d.

### 3.2. Release Profiles

**Figure 9:**
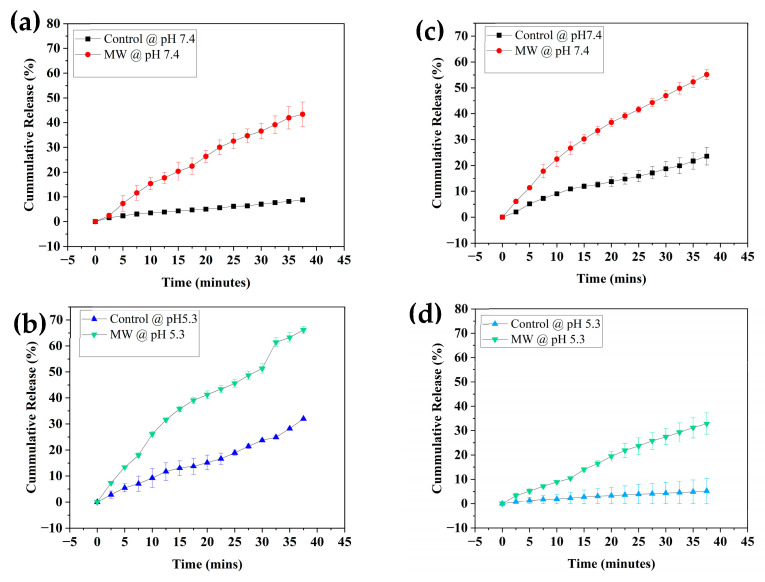
Doxorubicin-release profiles from (**a**) MIL-53 at pH 7.4 with and without MW irradiation; (**b**) MIL-53 at pH 5.3 with and without MW irradiation; (**c**) Fe-BTC at pH 7.4 with and without MW irradiation; (**d**) Fe-BTC at pH 5.3 with and without MW irradiation.

Figure 9a–d depicts the in vitro pH/MW triggered release profiles of MIL-53(Al) and Fe-BTC, respectively. The experiments were conducted in triplicates and the presented data represent the averages and the associated standard deviation error bars. Figure 9a–d shows that both MOFs exhibited high responsiveness to MW exposure compared to their counterparts without MW irradiation. Notably, MIL-53(Al) demonstrated dual responsiveness to both lower pH and MW irradiation, a remarkable feature. This property is particularly significant in anticancer drug-delivery applications due to the acidic nature of the tumor microenvironment (TME), as previously discussed. The acidity leads to protonation, resulting in the breakdown of the MOF’s coordination bonds [15]. This pH responsiveness ensures that drug release can be tailored to specific conditions in cancerous tissues, enabling precise and targeted therapeutic interventions. Furthermore, the introduction of MW exposure significantly increased the release of DOX from both MIL-53(Al) and Fe-BTC. This effect can be credited to the induction of heat through MW irradiation. Microwave irradiation induces polar molecule rotation that results in the thermal and heating effects of the solution [63]. This increase in temperature contributed to the enhanced release of the encapsulated DOX within the MOF’s pores. The synergy between pH-triggered responsiveness and MW-induced heating provides an effective strategy for controlled drug release in response to both the tumor microenvironment and external stimuli [50].

**Figure 10:**
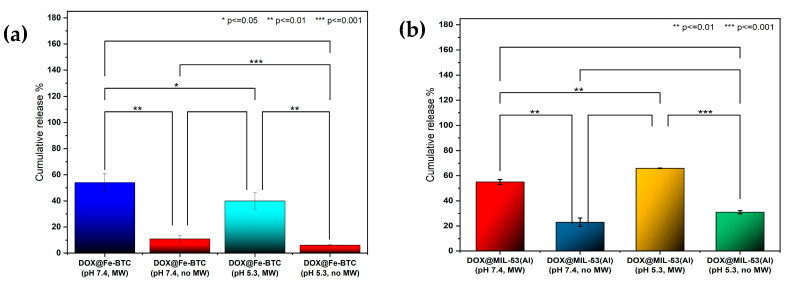
Comparison of in vitro DOX release, with and without microwave irradiation, for both (**a**) MIL-53 and (**b**) Fe-BTC. The error bars depict the standard deviation from three replicates, while the data points signify the average of these three independent measurements. Statistical significance was determined using ANOVA with Tukey’s method, denoted by *p*-values.

Figure 9a–d demonstrates the drug-release profiles for DOX from MIL-53(Al) and Fe-BTC, respectively. Exposure to MW irradiation at a frequency of 2450 MHz and a power of 200 W after 20 cycles (50 min) improved the drug-release efficiency by 32% at pH 7.4 for MIL-53(Al) and by 34% at pH 5.3 (Figure 9a,b). On the other hand, at a pH of 7.4, the improvement was more pronounced in the case of Fe-BTC than in the case of MIL-53(Al). The release % after 20 cycles was observed to be around 40% at pH 5.3 because of MW exposure, in contrast to only 6% without MW. While at pH 7.4, the release % reached up to 54% with MW and 11.4% without (Figure 9c,d). The *p*-value, determined using the two-tailed T-test, comparing experiments at pHs of 5.3 and 7.4 was calculated to be less than 0.05. Figure 10a,b represent the *p*-value summary for the maximum release after 40 min of MW irradiation for both MIL-53 and Fe-BTC MOFs, respectively. The preliminary results show clear enhancement in the MW release, regardless of the pH. The drug was not released even at lower pH values in the absence of an external MW stimulus.

### 3.3. Reactive Oxygen Species (ROS)

Reactive oxygen species (ROS) are generated as products of biological reactions and play an important role in supporting the oxidative processes in the body. In anticancer systems, ROS generated at the tumor site are used for targeted treatment by producing oxidative stress. MOFs are ideal candidates for such applications owing to their non-toxicity, porosity, size, and several other properties conducive to ROS [17,64].

**Figure 11:**
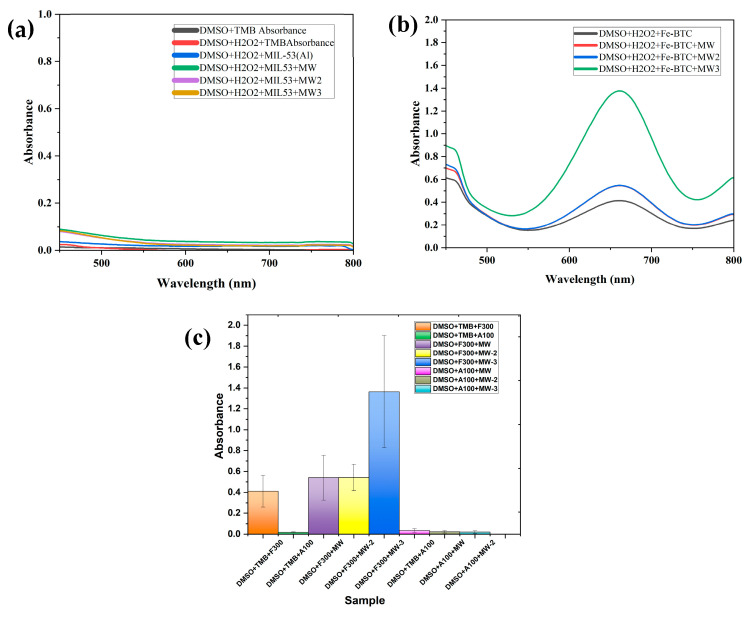
Absorption spectrum of DMSO alone compared with the absorption spectra of DMSO with H_2_O_2_ and (**a**) MIL-53; (**b**) Fe-BTC with and without MW irradiation; (**c**) bar-chart depicting standard deviations of different ROS samples.

The absorption spectra of DMSO in MIL-53(Al) and Fe-BTC are shown in Figure 11a,b, respectively. The DMSO and TMB mixture exhibited no peak at 652 nm, indicating no ·OH generation. This changed with the addition of H_2_O_2_ and Fe-BTC. Unlike MIL-53(Al), Fe-BTC catalyzed H_2_O_2_ and produced ·OH under 20s of MW irradiation, as shown in Figure 11a. The color change is associated with the formation of oxidized 3,3′,5,5′-tetramethylbenzidine (TMB), a product of the reaction between ·OH and TMB [52]. On the other hand, no color change was observed for MIL-53(Al). The absence of net ·OH radicals generated in the case of MIL-53(Al) could be attributed to the benzene dicarboxylic acid (bdc) ligand present in the MIL-53 framework. Bdc is identified as an ·OH scavenger. Previous studies have reported that bdc reacts with ·OH radicals to form hydroxy cyclohexadienyl. Bdc or hydroxylated bdc may be formed as end products by dismutation in this pathway; hence, the MOF framework may be conserved during the ·OH scavenging process by bdc. MIL-53(Al) is also highly stable and hydrolysis resistant in neutral and acidic solutions; this could be attributed to the strong chemical bonding between aluminum atoms in the secondary building units and oxygen atoms. Moreover, the aromatic walls in MIL-53(Al) render the structure hydrophobic and prevent the dissolution of the framework. All of these properties combined could be the reason no ROS generation was detected for MIL-53(Al) [65,66].

**Figure 12:**
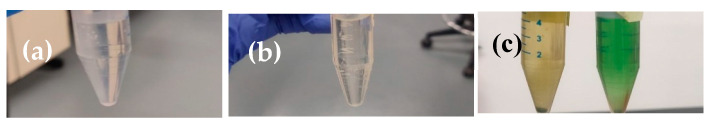
ROS experiment on (**a**) DMSO after MW irradiation; (**b**) DMSO + TMB + H_2_O_2_ post MW irradiation; (**c**) DMSO + TMB + H_2_O_2_ + A100/Fe-BTC post MW irradiation.

As illustrated in Figure 11b, the concentration of ROS increases with the addition of Fe-BTC, reaching its peak after MW irradiation. It was observed that the ROS generation slightly decreased after initial exposure to MW irradiation but gradually increased after the second cycle. However, it is essential to control ROS generation to avoid interfering with the pH-responsive property of the drug-delivery system. Figure 11c shows the standard deviation of the different ROS experiments. The *p*-value for Fe-BTC for ROS generation with MW irradiation was calculated to be 0.038, whereas for MIL-53(Al) it was 0.185, which proves the significance of the data collected for Fe-BTC. Figure 12 shows the color change before and after MOF addition and MW irradiation, with a green color change indicating ROS production.

### 3.4. Cell Viability and Cytotoxicity Tests

**Figure 13:**
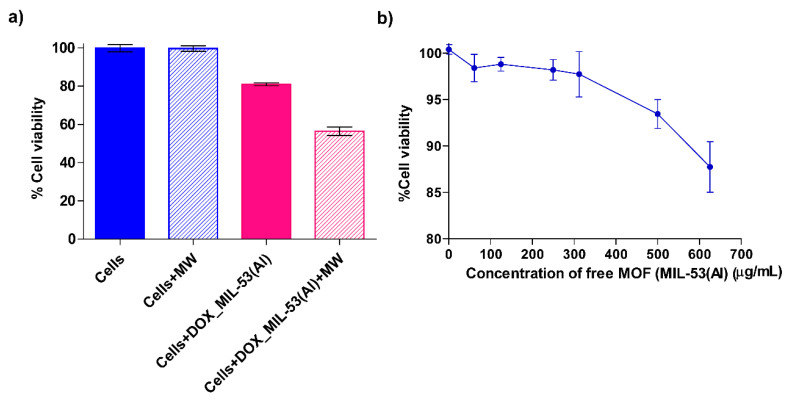
Cytotoxicity assays on Jurkat cell lines. (**a**) Impact of microwave treatment on doxorubicin release from MIL-53(Al) metal-organic frameworks (MOFs) and cell viability reduction in Jurkat cell lines. The test solutions (Cells + DOX_MIL-53(Al) + MW) were subjected to microwave treatment, leading to a statistically significant reduction in cell viability with *p* < 0.0001 compared to the untreated control data. One-way ANOVA was used for statistical estimation. Data in the figure represent means ± SD of three independent experiments. (**b**) Cytotoxicity studies with free MIL-53(Al). The graph shows the variation in viability of cells (Jurkat) with DOX-unloaded MIL-53(Al).

We conducted cytotoxicity assays using Jurkat cells by systematically exploring the effect of MW irradiation on different test culture solutions (DOX-loaded MIL-53(Al)). Our findings depicted in Figure 13a highlight a substantial decrease in cell viability for individuals exposed to MW-treated DOX-loaded MIL-53(Al). Specifically, the viability dropped to around 55% compared to the control group, which retained 80% viability without MW treatment.

This investigation sheds light on the cytotoxic effects caused by MW treatment, revealing the process of DOX release from MIL-53(Al) MOFs and its subsequent influence on the viability of Jurkat cell lines. Our results showed a substantial decrease in cell viability, supported by a remarkably low *p*-value of <0.0001 when compared to the control data without MW treatment, thus providing strong evidence of the effectiveness of MW treatment in facilitating the release of the therapeutic agent from MIL-53(Al) MOFs and its consequential influence on cell viability.

Furthermore, we conducted cytotoxicity evaluations involving unloaded MIL-53(Al) metal-organic frameworks (MOFs). This investigation explored how Jurkat cell viability was affected when exposed to MIL-53(Al) MOFs lacking DOX. The results of our MTT assay (Figure 13b) revealed consistently high cell viability even when subjected to different concentrations of the MOFs during a two-day incubation period. Even at a high concentration of 625 µg/mL, the MOFs exhibited exceptional biocompatibility, indicating their potential suitability for targeted tumor therapy aligning with what was reported before [67,68]. In conclusion, our findings highlighted the prolonged cell viability at various exposure durations, affirming the safe applicability of MW as a modality for triggering drug release from MOFs. These results collectively suggest the promising biocompatibility and safety profile of MIL-53(Al) MOFs, thereby positioning them as promising candidates for targeted cancer therapy and reinforcing the utility of microwave irradiation as a safe and effective modality for enhancing drug release from MOFs [69,70].

## 4. Conclusions

In this study, we investigated the utilization of metal-organic frameworks (MOFs) as cancer-drug-delivery vehicles to find a safer and more controlled drug-release mode to minimize side effects. We compared the Fe-BTC and MIL-53(Al) MOFs used in drug-delivery applications and studied microwaves (MW) as a stimulus for the controlled release of doxorubicin to be able to design a system that would reduce the side effects of the drug. We focused on the properties and application of MW as a stimulus to induce heat at the targeted location, which would consequently generate structural changes in MOFs for the controlled release of any drug encapsulated within.

The drug was effectively loaded in the MOFs, supported by the FTIR and EDX results. The release efficiencies varied, but the overall efficiencies of the MW experiments were higher than those of the control experiments, which proves that MW could be an effective stimulus for the release of DOX from Fe-BTC and MIL-53(Al) along with the pH of the tumor site. The characterization tests advocated for the stability of both MOFs before and after loading, which is an essential property in drug-delivery applications. Preliminary cell viability tests using the MTT assay validated the biocompatibility of Fe-BTC and MIL-53(Al), as well as the security of MW irradiation as a stimulus. Further viability and biocompatibility tests are required to confirm the preliminary results. In this study, it is concluded that Fe-BTC and MIL-53(Al) are MW sensitive, stable, and biocompatible, which makes them ideal drug-delivery-vehicle candidates for targeted delivery of doxorubicin at the tumor site.

## Figures and Tables

**Table 1:** BET results for MIL-53(Al) and Fe-BTC.

Sample	Pore Volume cm^3^/g	Pore Diameter Å	Surface Area BET m^2^/g
MIL-5(Al)	0.24	12.318	329.741
DOX@MIL-53(Al)	0.186	12.318	203.435
DOX@MIL-53(Al) + MW	0.055	17.656	22.811
Fe-BTC	0.168	6.159	247.801
DOX@Fe-BTC	0.066	6.159	81.402
DOX@Fe-BTC + MW	0.051	36.274	27.654

## Data Availability

The original contributions presented in the study are included in the article, further inquiries can be directed to the corresponding author.
